# Important Role of the Ihh Signaling Pathway in Initiating Early Cranial Remodeling and Morphological Specialization in *Cromileptes altivelis*

**DOI:** 10.3390/ani13243840

**Published:** 2023-12-13

**Authors:** Xiaoying Cao, Shunyun Deng, Quanyin Liu, Lisheng Wu, Xuan Zhuang, Shaoxiong Ding

**Affiliations:** 1 State Key Laboratory of Marine Environment Science, College of Ocean and Earth Sciences, Xiamen University, Xiamen 361102, China; 2 Xiamen Key Laboratory of Urban Sea Ecological Conservation and Restoration, College of Ocean and Earth Sciences, Xiamen University, Xiamen 361005, China; 3 Department of Biological Sciences, University of Arkansas, Fayetteville, AR 72701, USA

**Keywords:** skull morphology, frontal remodeling, osteoblast, gene expression

## Abstract

**Simple Summary:**

Adaptive radiation can lead to both morphological convergence and divergence. Understanding the molecular mechanisms driving these changes is essential for deciphering evolutionary relationships among species and the ecological implications of phenotypic variations in organisms’ natural habitats. The humpback grouper *Cromileptes altivelis* represents a distinctive coral reef fish classified in a monotypic genus due to its unique morphology. However, molecular phylogenetic analysis challenges this classification, suggesting its placement within the genus *Epinephelus*. Interestingly, the characteristic ‘sunken head and humpback’ does not manifest in *C. altivelis* during the early differentiation stages but gradually emerges during its developmental stages. Investigating the genetic basis of this phenotypic specialization feature is expected to provide evidence for resolving taxonomic controversies and advancing our understanding of speciation studies.

**Abstract:**

In this study, we identified the important contribution of frontal bone remodeling in shaping the ‘sunken head and humpback’ appearance in *C. altivelis*. Our investigation identified a developmental milestone at a total length of 5–6 cm, making the onset of its morphologic specialization in this species. A comparative analysis with closely related species reveals heightened activity in the frontal osteoblasts of the humpback grouper, potentially providing a physiological basis for its remodeling. Furthermore, our findings highlight that a significant upregulation in the expression levels of *Ihhb*, *Ptch1*, and *Gli2a* genes was seen in *C. altivelis* within the specified developmental stage, indicating an important involvement of the *Ihhb*-*Ptch1*-*Gli2a* signaling pathway in initiating the morphological specialization. We hypothesized that Ihh signaling could be attributed to shifts in mechanical stress, resulting from muscle traction on the frontal bone due to changes in swimming patterns during development. This study not only offers significant insights into unraveling the molecular mechanisms that govern phenotypic specialization and ecological adaptations in the humpback grouper but also serves as a valuable reference for studies on fishes with a controversial morphology and molecular phylogeny.

## 1. Introduction

The evolution of fish species through adaptive radiation has introduced intriguing conflicts between morphological and molecular classification systems [1]. In diverse ecological environments, closely related species, such as those in the family Bathydraconidae, *Heterochromis multidens*, and *Cichla temensis*, rapidly diverge into groups with distinct foraging strategies and physiological structures, resulting in significant phenotypic differences [1]. The research indicates that adaptive radiation not only drives morphological convergence but also contributes to morphological divergence, with specific mechanisms being a central focus of many researchers [2,3]. Unraveling these mechanisms is crucial to understanding the evolutionary relationships among species and the ecological implications of phenotypic variation in their habitats.

**Figure 1:**
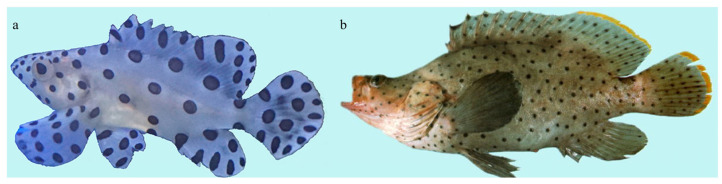
Morphological characteristics of *C. altivelis* adult and larvae: (**a**) adult (image from FishBase) and (**b**) larvae (without humpback).

The humpback grouper (*Cromileptes altivelis*, Valenciennes, 1828), highly valued for both its nutritional and ornamental qualities, is taxonomically classified within the genus *Cromileptes*, belonging to the family Epinephelidae in the order Perciformes [4]. However, the molecular phylogenetic analysis reveals its close affiliation with the *Epinephelus* branch, sharing close genetic relationships with numerous species in the genus *Epinephelus* [5,6]. Distinguished by features such as a sharper head and a pronounced bulge in its back, particularly behind the eyes (Figure 1a), *C. altivelis* stands apart from other groupers, leading taxonomists to classify it as a separate genus within the Epinephelidae family [4,7]. The discrepancies between the morphology and molecular phylogeny of *C. altivelis* suggest a plausible hypothesis that attributes these variations to differences in the evolutionary rate or expression of specific genes associated with the development of its unique phenotype. Consequently, *C. altivelis* emerges as a valuable subject for both phylogenetic and adaptive evolution studies.

**Figure 2:**
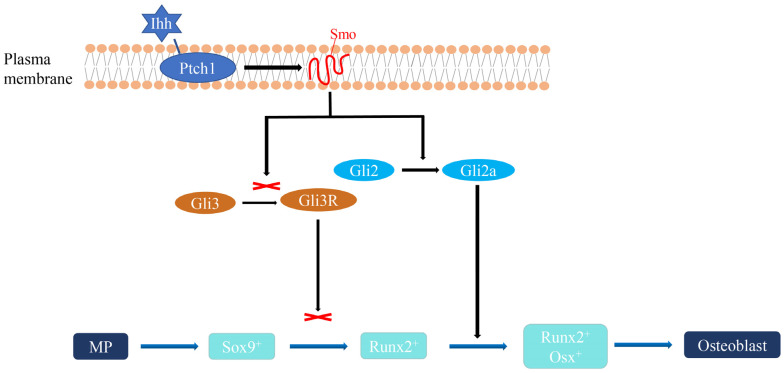
Ihh signaling pathway in regulating osteoblast differentiation (refer to Pan et al. [16] and Salhotra et al. [17] for further details).

In this study, we observed a close association between the distinctive phenotype of the humpback grouper and the development of its cranial skeleton. Key genes responsible for skeletal development likely contribute to the formation of these unique cranial structures. Among these genes, members of the Hedgehog (Hh) family hold particular importance [8]. The Hh family consists of a group of secreted protein ligands that play a crucial role in cell differentiation and development across both invertebrates and vertebrates [9,10]. In vertebrates, *Hh* genes are classified into three subgroups: *Dhh*, *Ihh*, and *Shh*. *Ihh*, as an upstream regulator of osteogenesis, plays a pivotal role in bone formation and development, particularly in osteoblast differentiation [11,12]. The *Ihh* ligand becomes active after modification, and in the absence of *Ihh*, the receptor Patched homologue (*Ptch1*) represses Smoothened homologue (*Smo*). When *Ihh* is present and binds to *Ptch1*, *Smo* repression is lifted, activating downstream transcriptional gene targets, such as the Gli family of transcription factors [13,14] (Figure 2). This activation, in turn, facilitates the differentiation of mesenchymal cells into osteoblasts. Furthermore, studies have demonstrated that the Ihh signaling pathway is both necessary and sufficient for craniofacial plasticity in teleost fish [8,15]. Therefore, although Ihh signaling is presumed to play a crucial role in early skeletal development in fish, the inquiry of whether it can influence the specific craniofacial formation in the humpback grouper through expression regulation remains a topic worthy of further investigation.

The ‘sunken head and hunchback’ morphology of *C. altivelis* is intriguingly absent during the early developmental stages after hatching, but it gradually emerges as development progresses. This observation leads us to propose that cranial development in *C. altivelis* undergoes anisotropic growth at a specific developmental stage, setting it apart from other closely related species and resulting in significant disparities in cranial morphology, ultimately giving rise to the characteristic ‘sharper mouth and humpback’ appearance. The Ihh signaling pathway, a central regulatory pathway involved in the differentiation of mesenchymal cells into osteoblasts, is likely to play a role in this developmental process. To test our hypothesis, we selected two closely related species (*E. coioides* and *E. lanceolatus*) as controls for this study. Firstly, we conducted a comparative analysis of morphological characteristics at different developmental stages in *C. altivelis* and its closely related species, identifying key components and developmental milestones contributing to the unique phenotype of *C. altivelis*. Secondly, we assessed osteoblast and osteoclast activity during the morphological specialization process, using histological sections and staining. Finally, we examined the relative expression levels of several key genes in the Ihh pathway at multiple developmental stages.

## 2. Materials and Methods

### 2.1. Sample Collection

*C. altivelis*, *E. coioides*, and *E. lanceolatus* larvae, each with a total length of about 2 cm, were collected from a commercial aquaculture farm in Hainan Province, China. At this stage, *C. altivelis* had not yet developed the ‘sharper mouth and humpback’ trait. All samples were reared in a water recirculating system with a salinity range of 29‰ to 32‰. They were fed with a formulated diet, and daily cleaning removed the residual bait and feces. The water temperature is controlled at 24–29 °C, with controlled lighting for 14 h. Water nitrate and ammonia nitrogen were regularly monitored, and 20–25% of the water was changed monthly to maintain a healthy environment. To identify the critical developmental stages and key differential skeleton of *C. altivelis*, we established four developmental stages for experimentation, as shown in Table 1. Each group consisted of three replicates, and each replicate contained 3 samples (for details, see Supplementary Table S1). Experimental fish were anesthetized using MS-222 (Sigma Aldrich Chemie GmbH, Schnelldorf, Germany).

### 2.2. Comparison of Bone Morphology

To investigate the potential factors contributing to the morphological specialization of *C. altivelis*, we conducted CT scans on individuals at various developmental stages. Subsequently, 3D reconstruction was carried out using Dragonfly (2022.2) software to facilitate a more comprehensive observation of skeletal changes in *C. altivelis*. Furthermore, bone staining and anatomy were performed on the three species of groupers in the first three developmental stages (S0, S1, and S2) to visually observe the change in skeletal morphology. However, in the last stage, staining was challenging due to the large size of the fish. Therefore, fish in the S3 stage were solely manually dissected. The bone-staining process [18] is as follows: specimens were fixed in 95% ethanol for 3 days; we then removed the skin and transferred the specimens to acetone for 1 day. Then, they were stained for 3 days in staining solution consisting of 1 vol 0.3% Alcian 8GX (Sigma) in 70% ethanol and 30% ethylic acid until the cartilage turned blue. Crystalline trypsin was used to digest the muscle, and the specimens were transferred to 1% KOH for 10 min at room temperature until the skeletons became clearly visible. After that, the specimens were fixed in staining solution consisting of 1 vol 0.1% Alizarin red (Sigma) in 95% ethanol and 0.5%KOH to stain the skeletons. For storage, specimens were transferred into 30%, 50%, 70%, and, finally, 100% glycerol. Combining these results, measurements were conducted on bones with significant differential changes before and after the morphological specialization of *C. altivelis* to identify key developmental stages in morphological transformations.

### 2.3. Quantification of Bone Sections

The morphological changes in the skeletal structure are primarily associated with the dynamic balance between osteoblasts and osteoclasts. To determine the variations in osteoblasts and osteoclasts during the humpback development in *C. altivelis*, key bones from the four developmental stages of *C. altivelis* and its closely related species were sliced and stained using enzymatic markers ALP (alkaline phosphatase) and TRAP (tartrate-resistant acid phosphatase) to quantify the osteogenic and osteoclastic activity. The samples were fixed with 4% paraformaldehyde and dehydrated with 30% sucrose to make 12–20 μm sections, using cryomicrotome (Leica CM1950, Wetzlar, Germany), and stained with ALP and TRAP staining kits (Solarbio, Beijing, China). We utilized ImageJ2 software to select areas of positive staining in the section image for grayscale mean analysis. For the black positive areas in ALP staining, we conducted mean grayscale measurements immediately after applying the inverted color treatment. In the case of the purple-red positive regions in TRAP staining, we employed the RGB stack and Split Channels functions within the software to separate them into multiple color channels. Subsequently, we selected the appropriate channels for inverse color measurements.

### 2.4. Sequence Analysis and qPCR

Based on the above results, we selected the IHH signaling pathway necessary for osteoblast differentiation, including *Ihhb* (the orthologues of *Ihh*), *Ptch1*, *Gli2*, and *Gli2a* genes, as the target genes in this study. We downloaded the gene sequences mentioned above for the humpback grouper and its closely related species, including *E. coioides*, *E. lanceolatus*, *Epinephelus fuscoguttatus*, *Epinephelus moara*, and *Epinephelus akaara*, from NCBI. We compared these sequences using MEGA [19] and conducted a manual check to confirm if there were any differences between the humpback grouper and its closely related species. For the quantitative analysis of the four genes in the frontal bones of humpbacked grouper at different developmental stages, we selected opercular bones from the same developmental period, as well as frontal bones from the closely related species *E. coioides* and *E. lanceolatus* at the same developmental period as the controls. The *EF-1α* gene of *C. altivelis* was chosen as an endogenous reference to normalize the starting quantity of RNA [20], and the primers for each gene are shown in Table 2.

The total RNA of all samples was extracted using the TRIzol (Thermo Fisher, Waltham, MA, USA) method and was reverse transcribed into first-strand cDNA, using the SMARTTM RACE cDNA Amplification Kit (Takara Bio, Inc. Dalian, China). qPCR was performed through Quant Studio TM 6 Flex Real-Time PCR System Software (ABI, Foster City, CA, USA) with SYBR green (Accurate Biotechnology, Changsha, China). Forty cycles of amplification were performed with the system above (5 s at 95 °C, 30 s at 60 °C, and 30 s at 72 °C). Relative gene expression was analyzed by using the comparative Ct method (2^−ΔΔCT^ method) [21]. 

### 2.5. Statistical Analysis

We presented the experimental data as mean (mean) ± standard deviation (SD). The Kolmogorov–Smirnov test and Levene’s test were used for tested the normality and equality of variances, respectively. We employed the one-way analysis of variance (ANOVA) and LSD tests to evaluate the extent of variation among the three species at specific developmental stages, as well as the degree of variation within each species across the four developmental stages. Student’s *t*-tests were applied to examine the level of genetic variation among different tissues of humpback grouper at specific developmental stages. In these analyses, *p* < 0.05 was considered a significant difference, while *p* < 0.001 was set as a highly significant difference. Data visualization was performed using GraphPad Prism 8.0 (GraphPad Software, San Diego, CA).

## 3. Results

### 3.1. Identification of Skeletal Elements and Developmental Node in Morphological Specialization of C. altivelis

**Figure 3:**
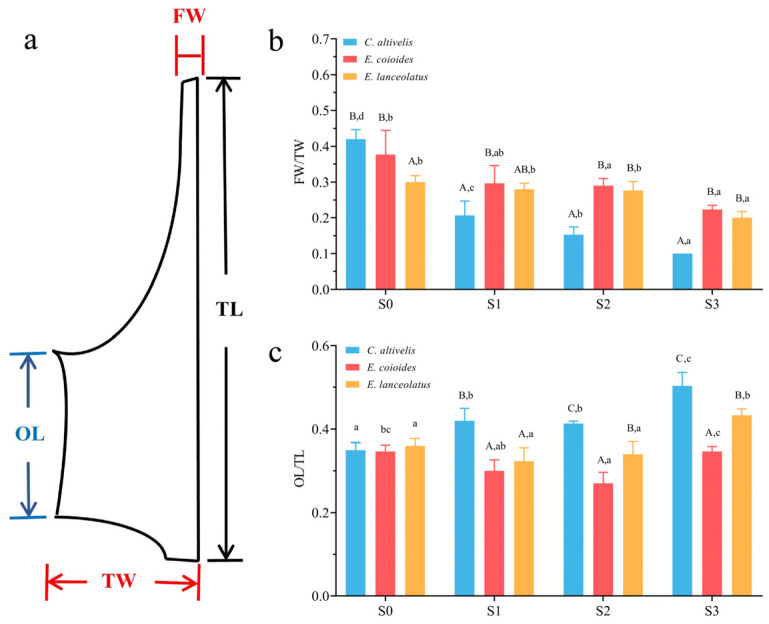
Comparison of frontal bone morphology in *C. altivelis* and its closely related species: (**a**) illustration of frontal bone measurements, (**b**) the ratio of FW/TW, and (**c**) the ratio of OL/TL. Uppercase letters indicate significant differences between different species at the same developmental stage (*p* < 0.05); lowercase letters indicate significant differences within the same species at different developmental stages (*p* < 0.05).

CT scans and 3D modeling revealed that the distinctive ‘sunken head and bulging back’ feature in *C. altivelis* primarily resulted from continuous remodeling and specialization of specific segments of the cranial skeleton (Supplementary Figure S1). The results from bone staining and dissection indicate that the skulls of the three species exhibited morphologically similar characteristics at the S0 (3–4 cm) stage (Supplementary Figures S2 and S3). However, at the S1 (5–6 cm) stage, *C. altivelis* had slight lateral growth at the top of the skull, although its ‘sunken head and humpback’ phenotype was not fully apparent (Supplementary Figures S2 and S3). At the S2 (7–8 cm) stage, a significant growth difference on the same skull component was observed in *C. altivelis* compared to closely related species. The skeleton on top of the skull no longer showed a curvature, and there was an apparent lateral stretch relative to its closely related species, implying a change in the morphology. The change became more pronounced at the S3 (9–10 cm) stage (Supplementary Figures S2 and S3). Among these features, the frontal bone, a key part of the humpback grouper with a concave head and a bulging back, undergoes notable morphological remodeling during specialization. To quantify these changes, we constructed a geometric model of frontal bone morphology and set four measurements, namely total length (TL), outer length (OL), fore width (FW), and total width (TW), to measure changes correspondingly (Figure 3a). The measurement results revealed that the FW/TW, representing the growth ratio of the width of the anterior and posterior segments of the frontal bone, did not exhibit significant differences among the three species at the S0 stage. However, the FW/TW ratio of the humpback grouper was significantly smaller than its closely related species during the S1–S3 stages (*p* < 0.05) and showed a decreasing trend at different developmental stages (Figure 3b). Similarly, the OL/TL, indicative of the longitudinal growth ratio, showed no significant differences at stage S0 stage but was significantly higher in *C. altivelis* compared to *E. coioides* and *E. lanceolatus* at the S1–S3 stages (*p* < 0.05, Figure 3c). Furthermore, the OL/TL ratio of *C. altivelis* was significantly higher in S1–S3 than in S0 (*p* < 0.05), while closely related species did not show the same morphological differences. Combined with anatomical observations, the anisotropic growth of the frontal bone in *C. altivelis* is characterized by elongation and anterior narrowing, particularly near the anterior end of the orbit.

### 3.2. Frozen Sections and Staining of Osteoblasts and Osteoclasts

**Figure 4:**
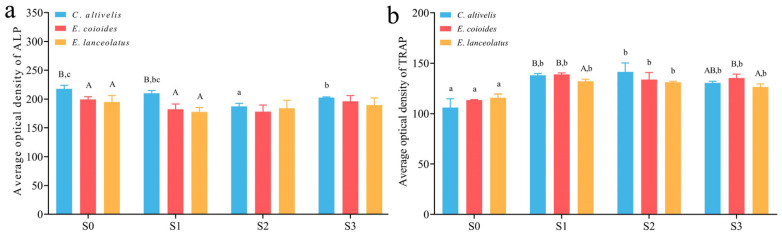
The ALP (**a**) and TRAP (**b**) optical density analysis in the frontal bone of three groupers. Uppercase letters indicate significant differences between different species at the same developmental stage (*p* < 0.05); lowercase letters indicate significant differences within the same species at different developmental stages (*p* < 0.05).

To understand the changes in osteoblasts and osteoclasts of the frontal bone remodeling in *C. altivelis*, the frozen section of the frontal bones of three groupers were stained with ALP and TRAP, which are markers of osteogenesis and osteoclasts, respectively. The average optical density of positive regions was calculated in the staining images. The results are as follows: At the S0 and S1 stages, the ALP-positive regions of the three groupers species were evenly distributed in all areas of the skeleton. However, at the S2 and S3 stages, there was a deeper ALP-positive region in the elongated frontal bone. The average optical densities of ALP-positive regions in *C. altivelis* were higher than those in *E. coioides* and *E. lanceolatus* (*p* < 0.05, Figure 4a) during the S0–S1 stages, indicating heightened active osteogenesis in *C. altivelis*. Furthermore, the areas with positive TRAP staining were predominantly concentrated near the pores in the bone tissue center of the three groupers, indicating increased osteoclastic activity in these regions. In comparison with related species, there was no significant difference in the average optical density of TRAP across the four developmental stages of *C. altivelis* (Figure 4b).

### 3.3. Expression Level of Ihh Pathway Genes

**Figure 5:**
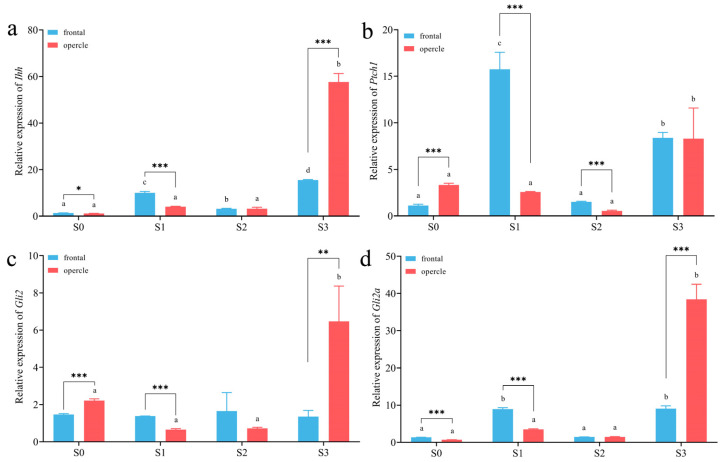
Quantitative analysis of Ihh signaling pathway genes in the frontal and opercular bone of *C. altivelis* at different developmental stages: (**a**) *Ihhb*, (**b**) *Ptch1*, (**c**) *Gil2*, and (**d**) *Gli2a*. Lowercase letters indicate significant differences in expression levels within the same tissue at different developmental stages; Asterisk indicates significant differences in expression levels between different tissues at the same developmental stage; * *p* < 0.05; ** *p* < 0.01; *** *p* < 0.0001.

We found that the *Ihhb*, *Ptch1*, *Gli2*, and *Gli2a* gene sequences of the humpback grouper did not exhibit specific nucleotide variations compared to the closely related species. A quantitative analysis of *Ihhb*, *Ptch1*, *Gli2*, and *Gli2a* genes in the frontal bone and opercular bone at four developmental stages of humpback grouper revealed the following results (Figure 5a–d):

In the frontal bone, the expression patterns of *Ihhb*, *Ptch1*, and *Gli2a* genes remained consistent across the four developmental stages, featuring an initial increase, followed by a decrease, and then a subsequent increase. Specifically, at stages S1 and S3, *Ihhb*, *Ptch1*, and *Gli2a* exhibited significantly higher expression levels (*p* < 0.05; Figure 5a,b,d), while the expression level of the *Gli2* gene did not show significant differences across the four developmental stages (Figure 5c). In the opercular bone, there were no significant differences in the expression levels of the four genes during stages S0–S2. However, a significant upregulation was observed at stage S3 (*p* < 0.05). When comparing the same developmental stage, during the S0 stage, the expression levels of *Ihhb* and *Gli2a* genes in the frontal bone were significantly higher than those in the opercular bone (*p* < 0.05; Figure 5a,d), while *Ptch1* and *Gli2* were significantly lower in the frontal bone compared to the opercular bone (*p* < 0.001; Figure 5b,c). At the S1 stage, the expression levels of all four genes in the frontal bone were significantly higher than those in the opercular bone (*p* < 0.001). During the S2 stage, only the expression level of the *Ptch1* gene in the frontal bone was significantly higher than that in the opercular bone (*p* < 0.001), while the other three genes showed no significant differences between the two tissues. At the S3 stage, the expression levels of *Ihhb*, *Gli2*, and *Gli2a* genes in the frontal bone were significantly lower than those in the opercular bone (*p* < 0.001; Figure 5a,c,d), while the expression level of the *Ptch1* gene showed no significant difference (Figure 5b).

**Figure 6:**
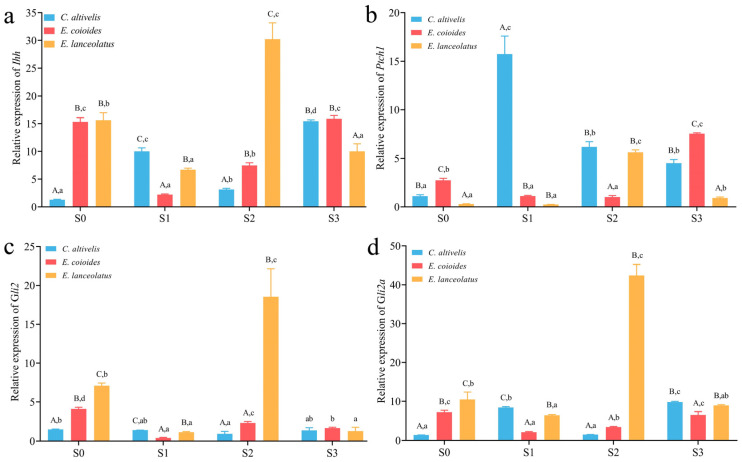
qPCR analysis of Ihh signaling pathway genes in the frontal bone of *C. altivelis* and its closely related species at different developmental stages: (**a**) *Ihhb*, (**b**) *Ptch1*, (**c**) *Gil2*, and (**d**) *Gli2a*. Uppercase letters indicate significant differences in expression levels between different species at the same developmental stage; lowercase letters indicate significant differences within the same species at different developmental stages.

Quantitative analysis of the four genes in the frontal bone tissue of *C. altivelis* and its two closely related species, *E. coioides* and *E. lanceolatus*, at different developmental stages yielded the following results (Figure 6a–d): During the S0 stage, the expression levels of *Ihhb*, *Gli2*, and *Gli2a* genes in humpback grouper were significantly lower than those in its related species (*p* < 0.05; Figure 6a,c,d). Additionally, the expression level of the *Ptch1* gene was significantly lower in *E. coioides* but higher in *E. lanceolatus* (*p* < 0.05; Figure 6b). At the S1 stage, the expression levels of all four genes in *C. altivelis* were significantly higher than those in its related species (*p* < 0.05; Figure 6a–d). At the S2 stage, the expression levels of *Ihhb*, *Gli2*, and *Gli2a* genes in *C. altivelis* were significantly lower than those in its related species (*p* < 0.05; Figure 6a,c,d), while the expression level of the *Ptch1* gene showed no significant difference with *E. lanceolatus* but was significantly higher than *E. coioides* (*p* < 0.05; Figure 6b). In the S3 stage, the gene expression levels of *Ihhb*, *Gli2*, and *Gli2a* in *C. altivelis* showed no significant difference compared to at least one of the related species, while the *Ptch1* gene was significantly lower in *E. coioides* but higher in *E. lanceolatus* (*p* < 0.05).

## 4. Discussion

The specialization of certain morphologies in fishes facilitates rapid speciation by enabling them to occupy various ecological niches within a relatively short time frame, ultimately fostering rich species diversity. One well-known instance of this phenomenon is the adaptive radiation of African cichlid fishes [22,23]. These cichlids have evolved to occupy distinct ecological niches primarily through the specialization of their jaw morphology, dentition, and digestive tract length, resulting in the differentiation into numerous cichlid species [23]. Similarly, the groupers are another example of adaptive radiation, diverging from its sister species approximately 40 million years ago [24]. This group boasts a wealth of diversity, with more than 170 species across 16 genera reported to date, many of which are sympatric [4,7,25,26]. *C. altivelis*, as one of them, inhabits lagoons and coastal coral reefs and is classified as a distinct genus due to its distinctive morphological feature—a ‘sharper head and pronounced bulge in its back’ [4,27]. Similar to the cichlids described above, this specialized morphology aids it in locating and preying on smaller fish, shrimp, crabs, and other invertebrates in the crevices of coral reef walls, increasing its predation success rate and providing favorable conditions for its rapid radiation. This study demonstrates that, compared to closely related species, the frontal bone of the humpback grouper exhibits allometric growth and morphological changes crucial to the formation of its ‘sunken head and humpback’ feature. The frontal bone, located in the anterior portion of the skull, forms the superior orbital wall and plays a significant role in the cranial structure, thereby influencing the cranial facial phenotype to some extent in animals [28,29]. For instance, in the Thalattosuchian Crocodylomorphs, partial evolutionary modifications of the frontal and nasal bones have led to distinct cranial facial phenotypes among species [28]. A further data analysis revealed that a total length of 5–6 cm was a critical developmental stage at which the frontal bone of humpback grouper begins to alter and remodel. The dynamic equilibrium between osteoblasts and osteoclasts plays a pivotal role in bone remodeling and growth [30,31,32]. Staining results for bone marker enzymes indicated that *C. altivelis* exhibits more active osteoblasts than its closely related species, suggesting that enhanced osteogenesis may provide the physiological basis for its morphological specialization.

Throughout the extensive evolutionary history of vertebrates, it is likely that rates of organ morphogenesis and genetic evolution did not remain constant or identical among some species [33]. Missense mutations and alterations in the expression of crucial genes at the molecular level can result in changes in skeletal morphology and phenotypic specialization [34,35,36]. For instance, studies involving the Scube signaling peptide have shown that mice with missense mutations in the exons of this gene exhibit abnormal bone morphology and altered parameters related to bone metabolism [36]. Similarly, missense mutations in the *BMP3* gene in domestic dogs have been linked to diverse craniofacial morphology [34]. Furthermore, research has demonstrated that changes in gene expression at specific stages in cichlid and heterochromatic medaka can lead to significant alterations in their craniofacial morphology [35,37]. Changes in the expression of genes such as *FGF* and *WNT* have also been shown to impact vertebrate skeletal morphology to some extent [38,39], highlighting the pivotal role of gene expression regulation in the specialization of skeletal morphology. The Ihh signaling pathway has been demonstrated to be a critical regulatory pathway for craniofacial remodeling in vertebrates [16,37], as well as for the proliferation and differentiation of various types of bone cells [17,32,40]. *Ihh* knockout mice exhibit smaller cranial bones [41]. Furthermore, its receptor gene, *Ptch1*, has shown differential expression in the jaws of distinct cichlid species with varying diets, correlating with their diverse maxillofacial structures [42,43]. In this study, we examined the coding sequences of *Ihhb* (the orthologues of *Ihh*), *Ptch1*, *Gli2*, and *Gli2a* genes in the Ihh pathway of the humpback grouper and its closely related species to rule out morphological specialization caused by missense mutations of these genes. A quantitative analysis revealed that the expression levels of *Ihhb*, *Ptch1*, and *Gli2a* genes in the frontal bones of the humpback grouper were significantly upregulated compared to both its own opercular bone tissue and the frontal bone of its closely related species during the critical developmental period (5–6 cm) for its morphological specialization. These findings suggest that the *Ihhb*-*Ptch1*-*Gli2a* signaling pathway plays an important role in the early formation of its specialized morphology.

Furthermore, the Ihh signal plays a crucial role in converting intracellular mechanical stress signals into biological signals [44]. Mechanical load is also considered one of the epigenetic factors influencing the craniofacial phenotypes of vertebrates [45]. It can lead to muscle attachments to bones experiencing traction and tension, resulting in bone deformation and elongation under the influence of axial forces. Nowlan et al. [46] utilized the finite element analysis to confirm that mechanical forces influence embryonic bone formation by regulating specific genes, with *Ihh* expression aligning with biophysical stimulation patterns. Additionally, another study showed that the application of cyclic tensile stress to chondrocytes led to increased *Ihh* expression and cell proliferation rates. These effects were halted when reagents blocking active stretch channels were introduced, resulting in inhibited cell proliferation [47]. Reports indicate that humpback groupers are suspended at the surface of the water during the early stage of hatching, while they prefer to hang upside down at the surface of the water or take shelter at the bottom of the pool during the juvenile stage [48]. It has been hypothesized that the humpback grouper may undergo a swimming pattern shift during development, causing the frontal bone of the head to be pulled by the muscles and the bone cells to sense this mechanical stress, triggering Ihh signals. Remarkably, the expression level of the *Ptch1* receptor gene in *C. altivelis* was more than 7-fold higher than that of other controls, significantly exceeding the expression levels of its upstream *Ihhb* gene and downstream *Gli2a* gene. This heightened *Ptch1* expression was shown to enable the formation of co-receptor complexes with an increased number of cell-surface molecules, enhancing its ligand-binding advantage and further intensifying the activity of this signaling pathway [49,50]. The *Gli2* gene did not exhibit significant differences across the four developmental stages. *Gli2* was inhibited by the sheath protein system (*Sufu*) in the non-activated state, and dissociated from *Sufu* after being activated by the *Ihh*-*Ptch1* signal. It then entered the nucleus in the form of full-length activation *Gli2a* to initiate the transcription of target genes [50]. These findings suggest that the *Ihh*-*Ptch1* signal likely regulates the transcription of target genes by influencing the degree of dissociation between *Gli2* and *Sufu* promoted by the intensity of signal transduction. It then regulates the differentiation and cell proliferation rate of skeletal cells, initiating the morphological specialization process, which is independent of the initial amount of *Gli2*.

## 5. Conclusions

In this study, our comparative analysis of skeletal characteristics in *C. altivelis*, in contrast to closely related species, highlights the important role of the frontal bone in driving the morphological specialization of *C. altivelis*. Notably, the remodeling specialization of the frontal bone in humpback grouper initiates at the 5–6 cm stage. We hypothesize that alterations in swimming patterns induce mechanical stress in skeletal cells, subsequently triggering Ihh signaling and initiating the morphological specialization process through the *Ihhb*-*Ptch1*-*Gli2a* regulatory pathway. This work provides important clues for further studies on the molecular mechanisms of the phenotypic specialization and ecological adaptations of the humpback grouper, as well as evidence for clarifying of its taxonomic controversy and speciation studies.

## Figures and Tables

**Table 1:** Setting of developmental stages of *C. altivelis* and its closely related species.

Developmental Stage	Total Length
S0	3.0–4.0 cm
S1	5.0–6.0 cm
S2	7.0–8.0 cm
S3	9.0–10 cm

**Table 2:** Primers for qPCR analysis.

Primers	Sequence (5′-3′)	Amplicon Seizes (bp)
*Ihhb*-F	GGGTAGAGGCTATGGCAAGAG	183
*Ihhb*-R	TTGAAGATGATGTCGGGGTTGTAG	
*Ptch1*-F	CGCTCCACCTACAACATCTCAC	118
*Ptch1*-R	TTCTATCTTTCCACCGCCACT	
*Gli2*-F	AGAACGGTAACTCCACTTATCCAC	198
*Gli2*-R	TGTGCTGCTCTGAAATCATCACC	
*Gli2a*-F	CGAAGCGACGCACCTCCTA	94
*Gli2a*-R	CCTCAATCTTGCCATCCACA	
*EF-1α*-F *EF-1α*-R	CAACTTCAACGCCCAGGTCA CTCATGTCACGCACAGCAAAA	276

## Data Availability

Data are contained within the article and Supplementary Materials.

## Supplementary Materials

The following supporting information can be downloaded at https://www.mdpi.com/article/10.3390/ani13243840/s1, Table S1: Sample information; Figure S1: The skull CT scan and 3D reconstruction of *C. altivelis* at different developmental stages: (a) 3 cm, (b) 5 cm, (c) 8 cm, (d) 10 cm, and (e) 20 cm; Figure S2: Transparent staining of *C. altivelis* and closely related species at S0–S2 developmental stages; Figure S3: The frontal bone of *C. altivelis* and closely related species at different developmental stages: (a) S0 (3–4 cm) stage, (b) S1 (5–6 cm) stage, (c) S2 (7–8 cm) stage, and (d) S3 (9–10 cm) stage; left, *C. altivelis*; middle, *E. coioides*; right, *E. lanceolatus*.
