# Supplementary material for: Important Role of the Ihh Signaling Pathway in Initiating Early Cranial Remodeling and Morphological Specialization in Cromileptes altivelis
Source: Animals (Basel). 2023 Dec 13;13(24):3840. doi: 10.3390/ani13243840 (PMC10740873; doi:10.3390/ani13243840)
Supplement: Supplementary file 1 [file animals-13-03840-s001.zip › animals-2648221-supplementary.pdf]

Supplementary Material for:

Important Role of the Ihh Signaling Pathway in Initiating Early Cranial Remodeling and Morphological Specialization in *Cromileptes altivelis*

Xiaoying Cao [1], Shunyun Deng [1], Quanyin Liu [1], Lisheng Wu [1,2], Xuan Zhang[3] and Shaoxiong Ding [1,2,*]

[1] State Key Laboratory of Marine Environment Science, College of Ocean and Earth Sciences, Xiamen University, Xiamen 361102, China

[2] Xiamen Key Laboratory of Urban Sea Ecological Conservation and Restoration, College of Ocean and Earth Sciences, Xiamen University, Xiamen 361005, China

[3] Department of Biological Sciences, University of Arkansas, Fayetteville, AR, United States

* Correspondence: sxding@xmu.edu.cn (S.D.)

Table S1 Sample information

Species	Sample	Total length	Wight
C. altivelis	CraltS001-S030	3.0-4.0 cm	1.0-1.8 g
	CraltS101-S130	5.0-6.0 cm	2.3-2.9 g
	CraltS201-S230	7.0-8.0 cm	7.8-9.5 g
	CraltS301-S330	9.0-10.0 cm	14.5-17.8g
E. coioides	EpcoiS001-030	3.0-4.0 cm	0.9-1.7 g
	EpcoiS101-130	5.0-6.0 cm	2.0-2.9 g
	EpcoiS201-230	7.0-8.0 cm	8.0-10.0 g
	EpcoiS301-330	9.0-10.0 cm	15.6-20.4 g
E. lanceolatus	EplanS001-030	3.0-4.0 cm	0.8-1.9 g
	EplanS101-130	5.0-6.0 cm	2.1-3.0 g
	EplanS201-230	7.0-8.0 cm	7.5-10.6 g
	EplanS301-330	9.0-10.0 cm	16.9-20.5 g

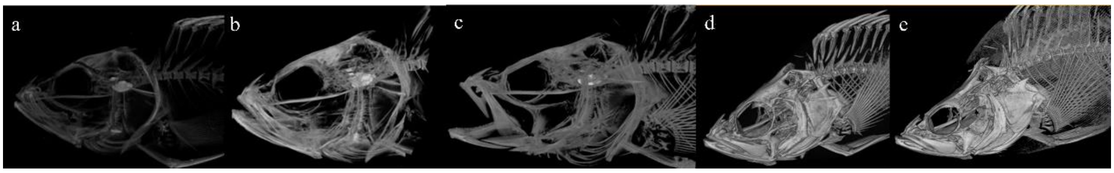

Figure S1. The skull CT scan and 3D reconstruction of *C. altivelis* at different developmental stages. a: 3cm; b: 5cm; c: 8cm; d: 10cm; e: 20cm.

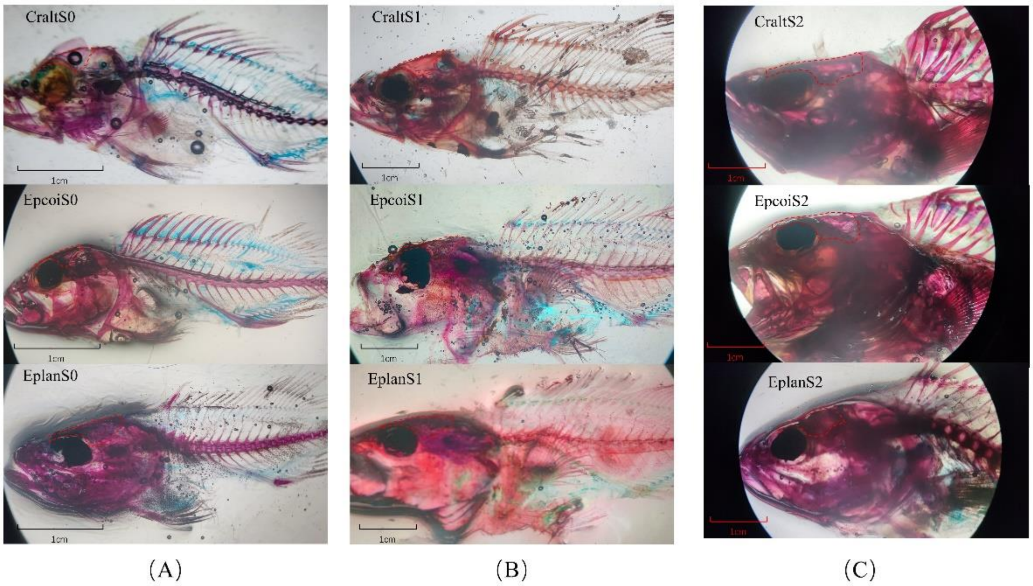

Figure S2. Transparent staining of *C. altivelis* and closely related species at S0-S2 developmental stages. Cralt: *C. altivelis*; Epcoi: *E. coioides*; *Eplan*: *E. lanceolatus.*

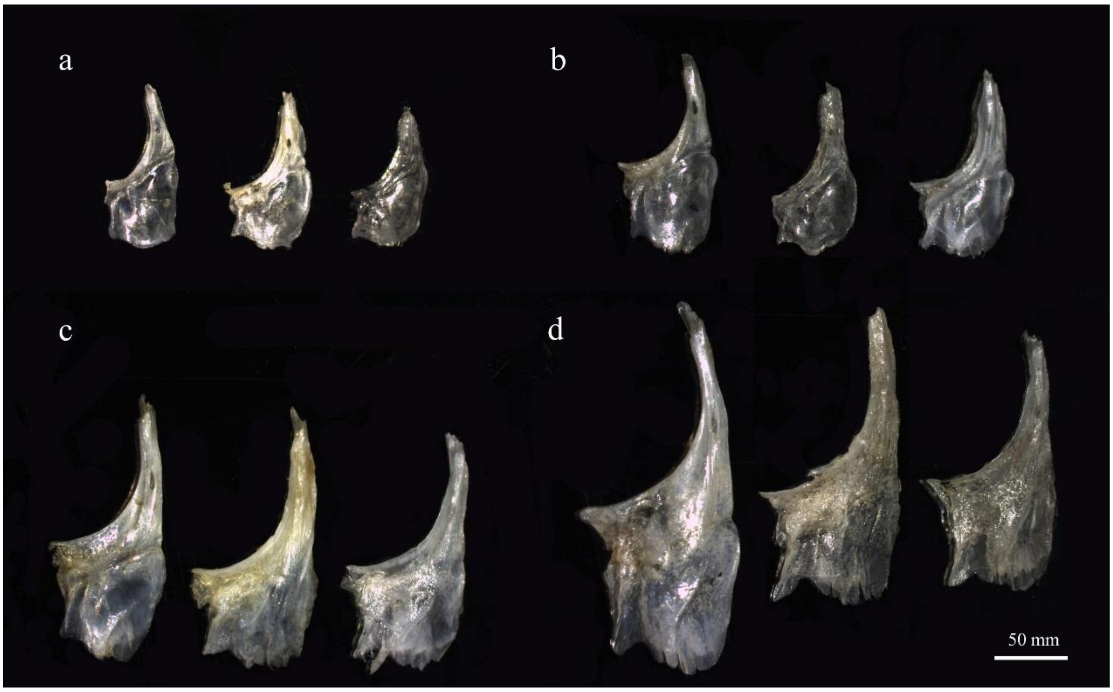

Figure S3. The frontal bone of *C. altivelis* and closely related species at different developmental stages.

a: S0 (3-4cm) stage; b: S1 (5-6cm) stage; c: S2 (7-8cm) stage; d: S3 (9-10cm) stage; left: *C. altivelis*; middle: *E. coioides*; right: *E. lanceolatus*.
